# Secundum Atrial Septal Defect With Early Presentation of Eisenmenger Syndrome and Right-Heart Failure: A Rare Case Report and Literature Review

**DOI:** 10.7759/cureus.8980

**Published:** 2020-07-03

**Authors:** Jahanzeb Malik, Umar Ikram, Ahmed Kamal, Ahsan Khalid, Tayyaba Zahid

**Affiliations:** 1 Cardiology, Rawalpindi Institute of Cardiology, Rawalpindi, PAK

**Keywords:** atrial septal defect, congenital heart disease, right to left shunt, eisenmenger, right heart cath, right heart failure

## Abstract

Eisenmenger syndrome, the most advanced form of pulmonary arterial hypertension (PAH), poses a considerable risk to the survival and quality of life of patients. It is more commonly seen in large intra-cardiac defects like ventricular septal defects (VSD) or patent ductus arteriosus (PDA), and rarely in atrial septal defects (ASD). Early diagnosis is the single most important step in the definitive management of the condition; otherwise, only conservative treatment can be offered. In this report, we present the case of a 20-year-old female patient diagnosed with Eisenmenger syndrome secondary to a large secundum ASD. The patient responded well to medical treatment.

## Introduction

Eisenmenger syndrome refers to the sequelae of untreated congenital heart defects (CHD) that lead to pulmonary hypertension [1-3]. Due to prolonged left-to-right shunt caused by a CHD, right-sided pulmonary pressures increase along with pulmonary vascular resistance, which causes shunt reversal leading to cyanosis [4]. CHDs like atrial septal defects (ASD) and ventricular septal defects (VSD) are characterized by high pressure in pulmonary vasculature leading to the sequela if untreated, but ASD usually presents in the fourth or fifth decade of life with Eisenmenger [5]. At this point, the defect is generally not operable. Eisenmenger syndrome itself has several grave complications like thromboembolism, infective endocarditis, heart failure, and arrhythmias [5]. It is a World Health Organization (WHO) risk class IV condition in pregnant females, and pregnancy is contraindicated according to the European Society of Cardiology guidelines [6]. Eisenmenger syndrome is more common with large VSD or patent ductus arteriosus (PDA) than with ASD [7]. Although its prevalence has decreased in the western countries [8], it remains a serious predicament in Pakistan where timely intervention still falls short. We present here a newly diagnosed case of a large ASD that was managed at our institute.

## Case presentation

**Figure 1:**
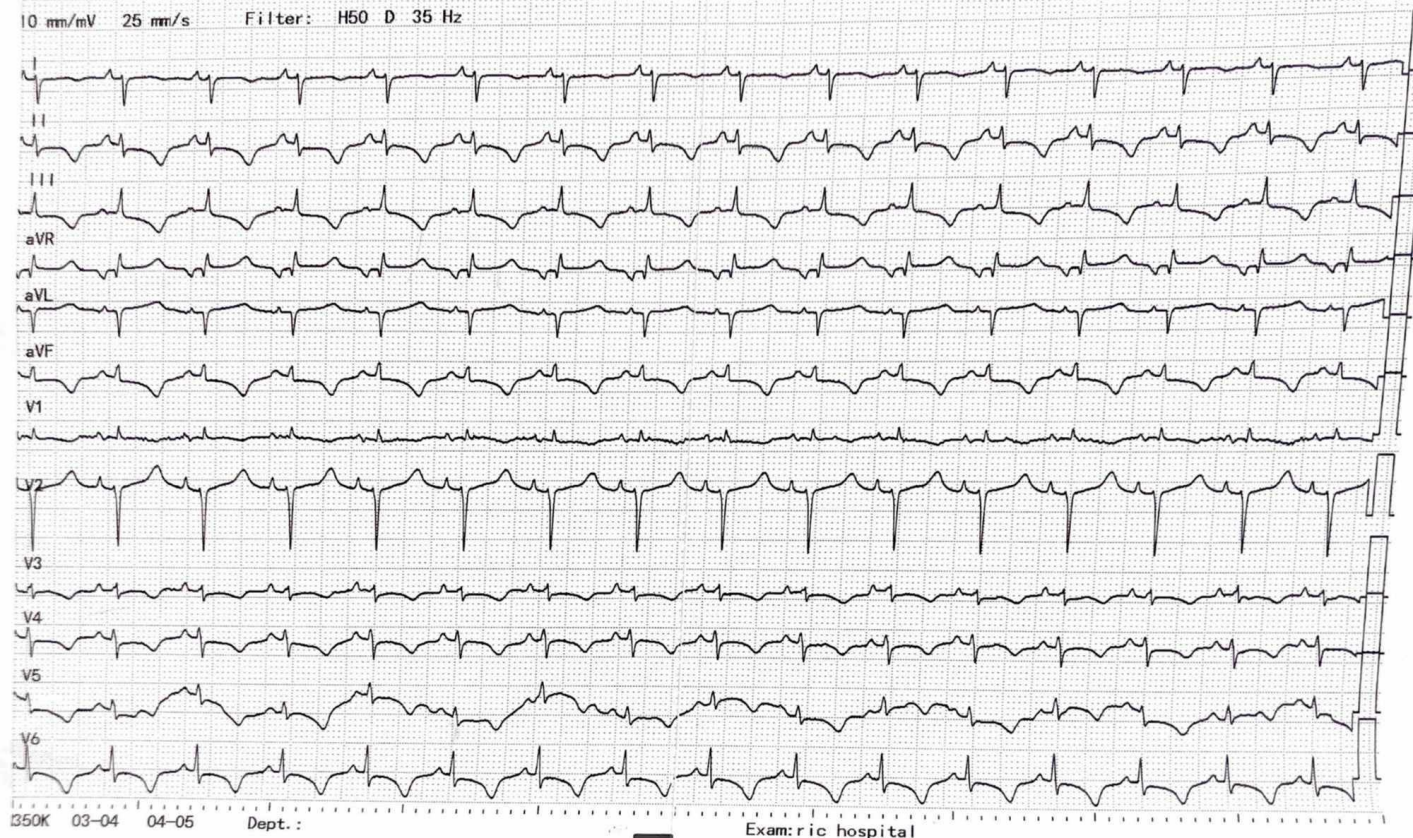
ECG showing right bundle branch block ECG: electrocardiogram

**Figure 2:**
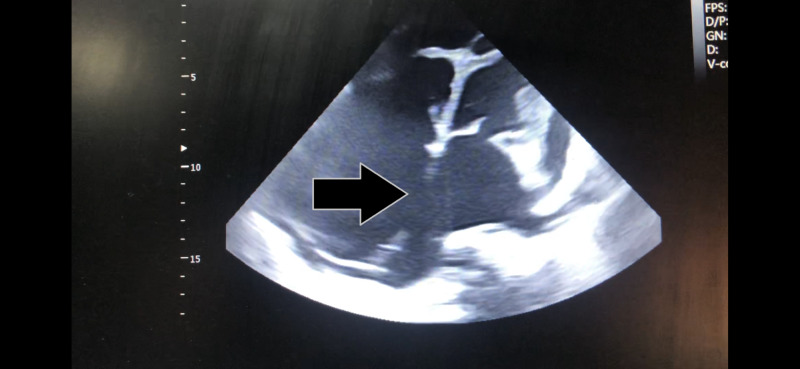
Large secundum atrial septal defect with a dilated right heart (black arrow)

A 20-year-old female, with a known history of heart murmur and non-compliant follow-up appointments, presented with dyspnea on exertion. She was admitted to the hospital with new-onset chest pain and shortness of breath. The pain was central and retrosternal, which was exacerbated when lying flat but improved while sitting. Her oxygen saturation was 83% and she was hypoxic. On physical exam, she had central cyanosis, jugular venous distention, parasternal heave, and a loud pan-systolic murmur. There was gross abdominal distension with ascites, and pedal edema with livedo reticularis. Her complete blood count revealed high hemoglobin of 18 g/dl, whereas liver function tests were mildly deranged with high alanine transaminase levels of 320 U/L and high gamma-glutamyltransferase levels of 60 U/L with borderline low albumin (2 g/dl). An electrocardiogram (ECG) showed a normal and regular heart rate with the right axis deviation as well as the right bundle branch block (Figure 1). The diagnosis was made on transthoracic echocardiography. It revealed a large ASD measuring 5.2 cm, severe dilation of right atria and ventricle, and severe tricuspid regurgitation with a mean pulmonary artery pressure of 110 mmHg (Figure 2; Video 1).

There was a shunt reversal on the Doppler assessment of the defect. On right-heart catheterization, there was 10% oxygen step-up from superior vena cava to right atrium indicating an ASD. Effective pulmonary blood flow to systemic blood flow (Qp/Qs) was 1, showing a bidirectional shunt and Eisenmenger physiology. Pulmonary artery pressure was increased (80 mmHg) with high pulmonary vascular resistance (9.2 Wood units) and a mean wedge pressure of 3 mmHg. The patient's prognosis was predicted using a six-minute walk test. It was about 260 meters, less than 50% of the predicted distance. She was classified as New York Heart Association (NYHA) Class III and started on sildenafil tablet 20 mg every eight hours and tablet bosentan 62.5 mg every 12 hours for four weeks. Her acute congestive symptoms were controlled using digoxin 0.25 mg daily, spironolactone 50 mg every 12 hours, and metoprolol 25 mg every 12 hours. Her symptoms improved within three days of hospital admission and were followed up after 30 days. She no longer felt dyspnea on exertion and her NYHA Class improved to Class II. Her six-minute walk test doubled to 500 meters.

**Video 1:** Secundum atrial septal defect with two-dimensional and Doppler assessment

## Discussion

After the bicuspid aortic valve, ASD is the most common adult CHD [9]. Approximately 70% of the ASDs are of the secundum type [9]. Most patients with ASD become symptomatic in the third or fourth decade of life with pulmonary hypertension progressing to the Eisenmenger phenomenon; however, our patient was relatively young to have developed severe right-heart failure and shunt reversal. Although the late presentation of ASD can occur due to the asymptomatic nature of the disease, other factors may be responsible in third world countries like Pakistan. They include poverty, limited access to medical facilities, inadequate medical expertise and diagnostic, and belief in unorthodox medicine.

Based on published literature, Eisenmenger syndrome is a rare complication of ASD. It occurs in less than 5% of patients and its development requires genetic predisposition [10]. It is considered to be a multisystem, progressive disorder that may present as fatigue, central cyanosis, digital clubbing, syncope, hemoptysis, and heart failure as the disease progresses. In patients with ASD, it is prudent to suspect Eisenmenger when they are cyanosed at rest due to large unrestricted defects like in the case of our patient. The diagnostic workup should be thorough. History and physical exam are the cornerstones, with the help of cardiac imaging. Echocardiography is of paramount importance in assessing any CHD, as it is cheap, readily available, and non-invasive. Cardiac catheterization can be done to ascertain the degree of shunt and pulmonary vascular resistance but is rarely indicated in advanced cases where no invasive treatment is of benefit except palliation.

In advanced cases like ours, conservative treatment has been shown to be of some benefit [11]. Drugs like prostanoids, phosphodiesterase type-5 inhibitors, and endothelin receptor antagonists are targeted at modifying the course of endothelin dysfunction. Studies have demonstrated an improvement in hemodynamics, exercise capacity, and quality of life in Eisenmenger syndrome [12]. There have been reports of Eisenmenger syndrome with ASDs as large as 2.5 by 3.5 cm with a known history of a murmur in a Nigerian male, and older males with the right bundle branch block as well as right ventricular hypertrophy [13]. Barnes et al. concluded that the mutations of bone morphogenetic protein receptor 2 (BMPR2) are responsible for 25% of idiopathic pulmonary arterial hypertension (PAH) associated with a valvular defect [14]. The diagnosis is made by right-heart catheterization provided that the mean pulmonary arterial pressure at rest is at least 25 mmHg.

There are two approaches to management. As described by Nashat et al., safe closure is possible if the net left-to-right shunting is greater than 1.5 and pulmonary vascular resistance is less than 2.3 Wood units, which was not the case in our patient [15]. To our knowledge, elevated liver function tests have not been used as criteria for surgery. The conservative management focuses on monotherapy including endothelin receptor antagonists, phosphodiesterase-5 inhibitors, and prostanoids. In a meta-analysis of eight studies, it was shown that bosentan and phosphodiesterase-5 inhibitors improved exercise tolerance and reduced vascular remodeling in patients, at the cost of a few adverse effects [16].

It has been proposed that endothelial dysfunction plays a role in PAH, and hence bosentan is currently endorsed as the first-line treatment in patients with Eisenmenger syndrome in the WHO functional class III-IV [17]. Endothelin-1 is a powerful vasoconstrictor seen in patients with PAH. This plays a key role in the pathogenesis of PAH by fibrosis and inflammation. These effects are produced by either endothelin A or endothelin B receptors on which endothelin receptor antagonists act, selectively targeting either single or both receptors [18]. In our patient, the functional improvement was seen after only a few weeks of treatment, which in turn endorses that theory.

## Conclusions

Eisenmenger syndrome, a rare complication of ASD, is dictated by both the size of the defect and the absence of specific genes. The larger size is responsible for both right- and left-sided heart failure. In patients with a failure to satisfy the criteria, medical management has to be used to achieve optimal functional class and symptom relief.
